# Aberrant activation of adenine nucleotide translocase 3 promotes progression and chemoresistance in multiple myeloma dependent on PINK1 transport

**DOI:** 10.7150/ijbs.101850

**Published:** 2025-01-01

**Authors:** Ke Hu, Yue Lai, Jinfeng Zhou, Chaolu Hu, Shushan Guo, Hui Zhang, Guanli Wang, Qikai Zhang, Xuejie Gao, Zhuning Wang, Yujie Liu, Qilin Feng, Hongfei Yi, Yu Peng, Yifei Zhang, Xiaosong Wu, Haiyan Cai, Jumei Shi

**Affiliations:** 1 Department of Hematology, Shanghai East Hospital, Tongji University School of Medicine, Shanghai 200120, China.; 2 Department of Hematology, Shanghai Tenth People's Hospital, Tongji University School of Medicine, Shanghai 200072, China.

**Keywords:** Adenine nucleotide translocase 3, Multiple myeloma, Bortezomib resistance, Mitophagy

## Abstract

Chemoresistance is an important factor in multiple myeloma (MM) relapse and overall survival. However, the mechanism underlying resistance remains unclear. In this study, we identified adenine nucleotide translocase 3 (ANT3) as a novel biomarker and therapeutic target for MM progression and resistance to the proteasome inhibitor bortezomib (BTZ). The oncogenic functions of ANT3 in MM were verified using MM sensitive/drug-resistant cells, bone marrow tissues from patients with MM, orthotopic MM model, and subcutaneous tumor model. ANT3 knockdown impaired MM cell proliferation owing to a lack of cellular ATP levels, causing cell cycle arrest in the G0/G1 phase. Moreover, our study showed that ANT3 leads to BTZ resistance by promoting mitophagy. Notably, ANT3-mediated mitophagy is independent of its biological function as an ADP/ATP translocase. Mechanistically, ANT3 interacts with mitochondrial inner and outer membrane transporters, including Timm22 and Tomm20, thus restricting PINK1 import to the inner membrane of mitochondria. In this case, PINK1 is stabilized in the outer membrane of the mitochondria and recruits Parkin, resulting in mitophagy. Furthermore, targeted intervention with ANT3 combined with BTZ limited the growth of BTZ-resistant myeloma *in vivo*. This study identified ANT3 as a novel biomarker and therapeutic target for MM.

## Introduction

Multiple myeloma (MM) is the second most common hematological malignancy, typically progressing from the precursor stage known as monoclonal gammopathy of undermined significance (MGUS) to asymptomatic smoldering myeloma and eventually to symptomatic MM^1^. Since the approval of the proteasome inhibitor bortezomib (BTZ) for clinical use, it has been widely used and listed as a first-line chemotherapy regimen for MM owing to its high efficacy, safety, and compatibility with other drugs. However, approximately 60% to 85% of relapsed/refractory patients with MM still exhibit BTZ resistance^2^, ^3^. Clinical resistance largely limits the improvement of progression-free survival (PFS) and overall survival (OS), and the median survival time of patients with MM is currently less than 8 years. In addition, clinical studies have shown that patients with MM who are intolerant or resistant to BTZ have shorter response time and an average survival time of only one year^3^. Therefore, exploring the causes and mechanisms of BTZ resistance in patients with MM is urgently required to overcome BTZ resistance and improve the clinical treatment efficacy^4^.

Adenine nucleotide translocase 3 (ANT3), also known as ADP/ATP translocase 3, is a mitochondrial protein encoded by the SLC25A6 gene and comprises six transmembrane segments^5^. The ANT family is one of the most abundantly expressed mitochondrial membrane proteins, and its expression is tissue-specific and dependent on the cellular energy requirements. ANT3 is ubiquitously expressed, and its expression reflects the level of oxidative phosphorylation in tissues^6^. The translocation of adenine nucleotides between the mitochondria and cytoplasm is one of the most characteristic mitochondrial activities. The ANT family, a member of the mitochondrial transporter family, mediates the exchange of cytosolic ADP and mitochondrial ATP driven by mitochondrial membrane potential (MMP). In addition, ANT, together with the voltage-dependent anion channel (VDAC) and cyclophilin D (CyPD), forms mitochondrial permeability transition pore (mPTP), which is involved in regulating apoptosis 7-9. Therefore, the normal expression of ANT is crucial for maintaining normal mitochondrial function and cellular homeostasis.

Despite its important biological functions, the effects of ANT3 on cancer are poorly understood. Previous studies have shown that ANT3 is essential for the growth of cancer cells and that its silencing leads to cell stress responses^10^. In addition, ANT3 is a substrate of the heat shock protein HSPA9, which participates in the survival of patients with BRAF-mutated tumors^11^. Hao *et al.* suggested ANT3 as a potential biomarker for the diagnosis of HPV^+^ cervical cancer with good specificity and sensitivity^12^. Interestingly, ANT3 has been found to have different and even opposite functions in various cell lines, suggesting that its function is highly cell-type-dependent^13^, ^14^. cDNA microarray analysis revealed that after treating various cancer cell lines, including hematologic malignant cells (such as HL60 and K562), with various chemotherapeutic drugs (including paclitaxel and homoharringtonine), the expression level of ANT3 changed markedly^15^. These studies indicate that ANT3 may be the molecular basis for acquired drug resistance in cancer cells. However, the role and molecular mechanism of action of ANT3 in mediating drug resistance requires further study. In addition, the role of ANT3 in hematological malignancies has not been reported.

Mitophagy is a major pathway for mitochondrial quality control, and its activation or inhibition depends on various factors such as cancer type and stage of cancer development^16^, ^17^. Studies have shown that mitophagy dysfunction leads to progression and drug resistance in various cancers^18^, ^19^. The anticancer effects of most chemotherapeutic drugs for MM are attributed to the induction of apoptosis caused by oxidative stress and mitochondrial dysfunction, whereas mitophagy maintains myeloma cell homeostasis by rapidly removing damaged mitochondria^20^, ^21^. However, both positive and negative regulatory effects of mitophagy on drug resistance have been reported. Whether mitophagy is a protective mechanism that ensures the survival of resistant MM cells during BTZ treatment requires further investigation.

In this study, we found that ANT3 is highly expressed and plays a vital role in myeloma progression and BTZ resistance. This indicates that ANT3 may be a novel and potential target for combating cancer drug resistance in patients with MM. Notably, we revealed the underlying mechanism by which ANT3 mediates mitophagy to promote BTZ resistance.

## Materials and Methods

### Cell culture and reagents

Wild-type MM cells were cultured at 37°C with 5% CO2 in Roswell Park Memorial Institute (RPMI) 1640 medium (Gibco) supplemented with 10% FBS and 1% penicillin-streptomycin, while HEK293T cell line and stably transfected cells were cultured in Dulbecco's modified Eagle's medium (DMEM, Gibco). The human ARP-1 MM cell line was provided by Dr. Fenghuang Zhan (University of Iowa, Iowa City, IA, USA); NCI-H929, RPMI8226 and their resistant cells were kindly provided by Prof. Wen Zhou from Central South University. Primary bone marrow cells were donated by healthy donors and MM patients, all of whom had informed consent. The experiment was approved by the Review Board and Ethics Committee of Shanghai Tenth People's Hospital.

Oligomycin A was purchased from Selleckchem (Houston, USA); carbonyl cyanide 3-chlorophenylhydrazone (CCCP), chloroquine diphosphate (CQ) and carboxyatractyloside (CTRA) were purchased from TargetMol (Boston, USA); and bafilomycinA1, rapamycin and MG132 were purchased from Sigma-Aldrich (Missouri, USA).

### Plasmid construction, virus packaging and cell infection

Using RNA purified from HEK293T cells as a template, the ANT3 sequence was amplified via PCR and inserted into the EcoRI and Xhol sites of the GFP-labeled pCDH vector (Haro life, Shanghai) and Flag-labeled vector, and the Timm10/22 and Tomm20 sequences into the HA-labeled vector, all of which were ultimately confirmed by sequencing. ShRNA was purchased from Qingke Biotechnology Company (as shown in Table S1). HEK293T cells were transfected with the lentivirus plasmids together with two auxiliary vectors, pMD2G and psPAX2, at a 1:1:1 ratio using Lipofectamine 3000 reagent (Invitrogen, California, USA). The packaged virus was collected, filtered and concentrated to infect MM cells, and the stably expressed MM cells were screened with 2-4 µg/mL puromycin (Beyotime Institute of Biotechnology). The lentivirus carrying luciferase was purchased from Shanghai Genechem Co. for secondary infection, and screened with neomycin.

### Cell viability assay

MM cells (2×10^5^ cells/mL) were seeded into 96-well plates and treated with different reagents. Subsequently, 10 μL of Cell Counting Kit-8 (CCK-8) solution (Yeasen Biotechnology Co., Ltd, Shanghai, China) was added, and the mixture was incubated for another 2 hours at 37°C. The absorbance was subsequently measured on a microplate reader (BioTek, Winooski, USA) at 450 nm.

### RNA extraction and qRT-PCR assays

Total RNA was extracted using an RNA-Quick purification kit (ES Science, Shanghai, China) according to the standard protocol and reverse transcribed to complementary DNA (cDNA) using a PrimeScript RT reagent kit (Takara Bio Inc., Shiga, Japan). The samples were then analyzed on an ABI 7000 PCR detection system (ABI, Vernon, CA, USA), after which the relative expression levels were calculated. The sequences of the primers used in this study are shown in Table S2.

### Western blot

Total cell protein was extracted using lysis buffer (100 mM Tris HCl pH 6.8, 4% SDS, and 20% glycerol), and was subsequently separated via sodium dodecyl sulfate-polyacrylamide gel electrophoresis (SDS-PAGE). The proteins were transferred to nitrocellulose membranes, which was then blocked with 5% nonfat milk at room temperature for 1 hour. The proteins were incubated overnight with the indicated antibodies at 4°C, and then incubated with the corresponding secondary antibody at room temperature for 1 hour. The protein bands were ultimately detected using Odyssey dual-color infrared laser imaging system (LICOR, Lincoln, NE, USA). Primary antibodies against PINK1and HA were purchased from Cell Signaling Technology (Danvers, USA); ANT3, Flag, LC3, P62, CDK4, CDK6, Cyclin B, and Cyclin D1were purchased from Abcam (Cambridge, USA); and Parkin was purchased from Abmart (Shanghai, China).

### Immunofluorescence staining

A total of 4×10^5^ cells were seeded onto 12-well plates the previous day, followed by the incubation with MitoTracker (200 nM) for 20 min the next day. Then, the cells were collected, washed and dropped onto adhesive slides until complete adhesion was achieved. Fixed with 4% paraformaldehyde (PFA) for 10 min, and blocked with 3% bovine serum albumin (BSA) and 0.03% Triton X-100 in PBS for 30 min at room temperature (RT), cells were sequentially stained with primary antibodies at 4°C overnight and fluorescent dye-conjugated IgG secondary antibodies at RT for 2 hours. After being sealed with the anti-quenching sealing agent, images were acquired with Leica TCS SPE confocal microscope using a ×100 oil immersion objective. MitoTracker and LC3 puncta were measured by Fiji Image and at least 20 cells in each group were subjected to quantification.

### Determination of mitochondrial membrane potential

The mitochondrial membrane potential was detected with a JC-1 kit (Beyotime Institute of Biotechnology). MM cells were treated with oligomycin A or CTRA for 2 hours, after which JC-1 working solution was added, and the cells were incubated in a cell incubator for 20 min. After washing and resuspending, the proportion of positive cells was analyzed by flow cytometry.

### Transmission electron microscopy (TEM)

The cell precipitates were collected and fixed overnight at 4°C in 2.5% glutaraldehyde. Then, the samples were treated with 1% osmic acid solution, dehydrated by a gradient concentration of ethanol, and finally treated with pure acetone for 20 min. After penetration, the samples were embedded and heated at 70°C overnight, and finally sliced to 70-90 nm. The sections were dyed in lead citrate solution and a 50% ethanol saturated solution of uranyl acetate, dried, and observed via transmission electron microscopy.

### Cell proliferation assays

For 5-ethynyl-2′-deoxyuridine (EdU) cell proliferation assay, MM cells were collected and EdU was incorporated into DNA using an EdU kit (Beyotime Institute of Biotechnology) according to the protocol. For soft agar colony formation assay, MM cells were resuspended in DMEM containing 20% FBS to a density of 4×10^4^/mL. The diluted cell suspension was then mixed with 1/5-volume 1.66% agar with or without drug treatment at a certain concentration. The cells were plated into 12-well plates containing a 0.7% bottom agar layer. The whole incubation period lasted for 14 days, after which the colonies were stained with 0.1% crystal violet. The colony numbers were determined by a digital camera and counted by ImageJ.

### Cellular ATP analysis

Myeloma cells were lysed on ice and centrifuged at 12,000 g for 5 min at 4°C. The detection steps followed the instructions of ATP assay kit (Beyotime Institute of Biotechnology). All the operations were performed on ice. Luminance (RLU) was measured by luminometer (Promega). The protein content of each sample was simultaneously detected by the bicinchoninic acid (BCA) standard curve method.

### Cell cycle analysis

After fixation with 75% ice-cold ethanol at -20°C overnight, the cells were washed once with phosphate-buffered saline (PBS), and then stained with PI/RNase staining buffer (BD Biosciences) at 4°C for 30 min. The DNA contents of the myeloma cells were subsequently analyzed by BD FACS Canto II (BD Biosciences). The cell cycle distribution was evaluated by ModFit LT v3.1 (Verity Software House, Topsham, ME).

### Study on mitochondrial function

For mitochondrial matrix calcium measurement, cells were collected and cultured with Rhod-2 AM working solution (Beyotime Institute of Biotechnology) for 30 min at 37°C. The stained cells were washed three times using Hank's Balanced Salt Solution (HBSS) and then analyzed by flow cytometer at excitation/emission wavelengths of 549/578 nm.

For the detection of mitochondrial superoxide, cells were collected and cultured with MitoSOX (Beyotime Institute of Biotechnology) for 30 min at 37°C. After PBS washing, the stained cells were analyzed by BD FACS Canto II (BD Biosciences).

### Co-immunoprecipitation (Co-IP) analysis

For Co-IP analysis, HEK293T and ARP-1 cells were transfected and collected according to the standard protocol. The cells were lysed in NETN solution (100 mM NaCl, 20 mM Tris-HCl, 0.5 mM EDTA, and 0.5% NP40) on ice for 30 min, followed by ultrasound-assisted lysis. The protein A/G beads (Biolinkedin, Shanghai, China) were incubated with the protein and corresponding antibodies at 4°C overnight in a rotator. After fully washing, the immunoprecipitant was resuspended in 2× protein sample buffer, and heated at 100°C for 6 min, and then detected by SDS gel electrophoresis with total protein simultaneously.

### Mouse model of human myeloma

All animal work was performed in accordance with the guidelines of Tongji University Institutional Animal Care and Use Committee under an approved protocol (NO: TJBB00223101). For subcutaneous tumor model, H929R cells (5×10^6^ in 50 μL of PBS) were mixed with an equal volume of matrix, and were injected subcutaneously into the dorsum of each 5-week-old Balb/c-nude mouse. The bortezomib groups were intraperitoneally injected with BTZ (1 mg/kg) on the 1st, 4th, 8th and 11th days, normal saline to control group. The tumor burdens (mm^3^) were measured every other day, and calculated using the following formula: V= length × width^2^/2. All the mice were euthanized at the end of the experiment, and the tumors were collected and subject to immunohistochemistry (IHC) staining.

For establishment of the myeloma orthotopic model, cyclophosphamide (100 mg/kg) was injected daily into 4-week-old NOD scid mice two days in advance. Then, ANT3-OE/EV RPMI8226-luc cells (8×10^6^ in 100 μL of PBS) were injected into the mice via the caudal vein. After tumor formation, *in vivo* imaging technology was used to determine the tumor burden at regular intervals. At the end of the experiment, the eyeballs of the mice were removed to obtain blood, separate the serum and to analyze alkaline phosphatase and lactic dehydrogenase level. Hematoxylin-eosin (HE) staining was performed on the left femur after separation and decalcification.

### Statistical analysis

The data are displayed as mean ± standard deviation (SD). One-way analysis of variance (ANOVA) or Student's t-test was used to determine the significance of multiple comparisons or two groups. ImageJ software (National Institutes of Health, Bethesda) was used to quantify the grayscale value of the immunoblots and the immunofluorescence intensity. GraphPad Prism 8.0 software was used for statistical analysis. *P*<0.05 was considered to indicate statistical significance.

## Results

### Aberrant ANT3 amplification is associated with MM progression and poor prognosis

**Figure 1:**
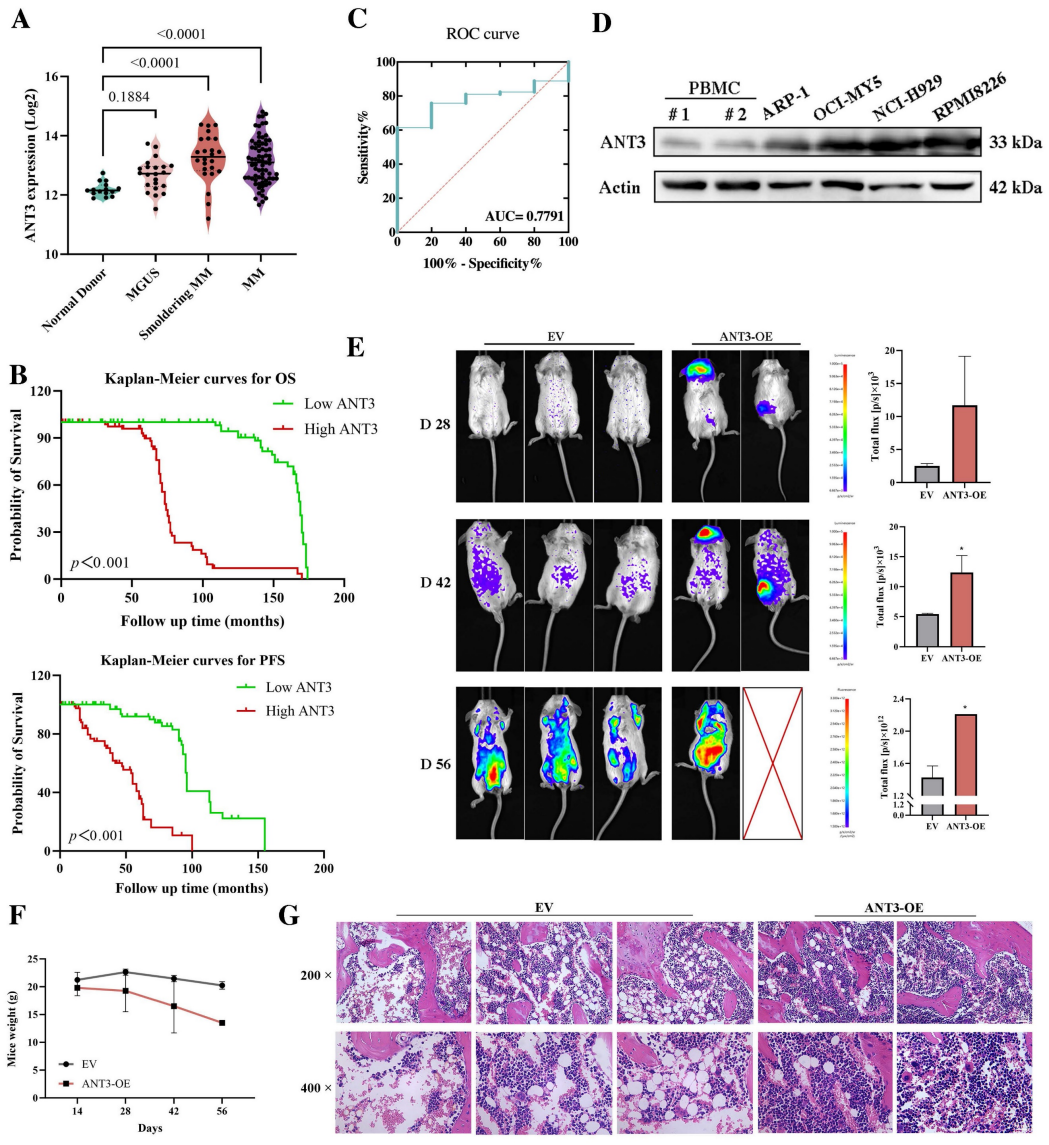
** ANT3 is associated with the occurrence, progression, and poor prognosis of multiple myeloma.** (**A**) Analysis of ANT3 expression in healthy donors, patients with monoclonal gammopathy of undetermined significance (MGUS), smoldering multiple myeloma (SMM) and MM by using publicly available data set from NCBI Gene Expression Omnibus (GEO) database (GSE6477). Statistics were calculated using Wilcoxon Rank Sum Test. (**B**) Kaplan-Meier curves of overall survival (OS) and progression-free survival (PFS) in MM patients related to ANT3 expression (red and green represent high and low expression levels of ANT3, respectively). The dataset was obtained from GEO database (GSE136324). Significant differences were calculated by Log-rank Mantel-Cox test. (**C**) Receiver operating characteristic curve (ROC) in 153 MM patients and 5 normal people by using publicly available data set from GEO database (GSE13591). Statistics were calculated using logistic regression. (**D**) Western blot showed the expression levels of ANT3 protein levels in MM cell lines compared with peripheral blood mononuclear cell (PBMC) of the normal donors, with Actin as a loading control. (**E**) The orthotopic model of myeloma was established by injecting RMPI8226 cells stably transfected with lentivirus-mediated human ANT3-cDNA (ANT3-OE) or the empty vector (EV) in the tail vein. The bioluminescence images and average intensity quantification in each group at 28, 42, 56 days after injection, * *P*<0.01. (**F**) Mice weight was recorded at 28, 42, 56 days after injection. (**G**) IHC analysis of femoral bone marrow, scale bars = 25 μM, original magnification: ×200 and ×400.

Although there have been no reports on the oncogenic effects of ANT3, its increased expression during the occurrence and progression of MM has attracted our attention. As shown in Figure 1A, compared with healthy donors, the expression level of ANT3 gradually increased during the development of myeloma, including MGUS and smoldering myeloma. We also explored whether ANT3 is related to the clinical biochemical features of patients with MM. According to the data set in the NCBI's Gene Expression Omnibus (GEO) database (GSE136324), patients with higher levels of β2-microglobulin (β2M), presence of cytogenetic abnormalities, and higher risk of international staging system (ISS) were more likely to have greater expression of ANT3 than patients without these risk factors (**Table 1**). Kaplan-Meier analysis was performed to evaluate the overall survival (OS) and progression-free survival (PFS) of patients with MM, who were classified according to the quartile level of ANT3 expression. Similarly, patients with higher ANT3 expression levels had worse outcomes (**Figure 1B**). The receiver operating characteristic curve (ROC) curve also demonstrated the value of ANT3 expression in determining MM prognosis, with certain sensitivity and specificity (**Figure 1C**). To further explore the expression level of ANT3 in pan-cancer, we searched the CCLE database (https://sites.broadinstitute.org/ccle), ANT3 expression levels in more than 1000 tumor cell lines from 24 tissues as well as in noncancerous cell lines were analyzed. The results showed that compared with non-cancerous cells, the expression of ANT3 in tumor cell lines was generally elevated (**Figure S1A**). Notably, the average expression level of ANT3 in all hematological malignancies included in the database, including myeloid leukemia, lymphoma, and myeloma cell lines, ranked at the forefront of all tissue-derived tumors. We also searched for GEPIA2 (http://gepia2.cancer-pku.cn/), which contains the expression profiles of 33 common human cancer types, excluding MM. We found that ANT3 was highly expressed in diffuse large B-cell lymphoma compared to that in normal tissues, which shares the same tissue origin as myeloma (**Figure S1B**). These results support the hypothesis that ANT3 expression is highly tissue-specific. Next, we examined the oncogenic effects of ANT3 in myeloma *in vivo*. NOD scid mice were injected with myeloma cells stably transfected with lentivirus-mediated human ANT3-cDNA (ANT3-OE) or the empty vector (EV) via the tail vein to establish models, and the tumor burden was recorded by *in vivo* imaging. In the ANT3-OE group, the tumor signal appeared earlier, grew faster, and the body weight decreased significantly, suggesting that high ANT3 expression contributed to MM occurrence and progression (**Figure 1E-F**). The levels of plasma cell infiltration, serum lactate dehydrogenase (LDH), alkaline phosphatase (ALP), and β2-microglobulin were higher in the ANT3-OE group than in the control group (**Figure 1G; Figure S1C**). These data further indicated that the expression level of ANT3 is positively related to the progression and severity of myeloma.

### ANT3 drives MM proliferation depending on its role in ADP/ATP translocation

**Figure 2:**
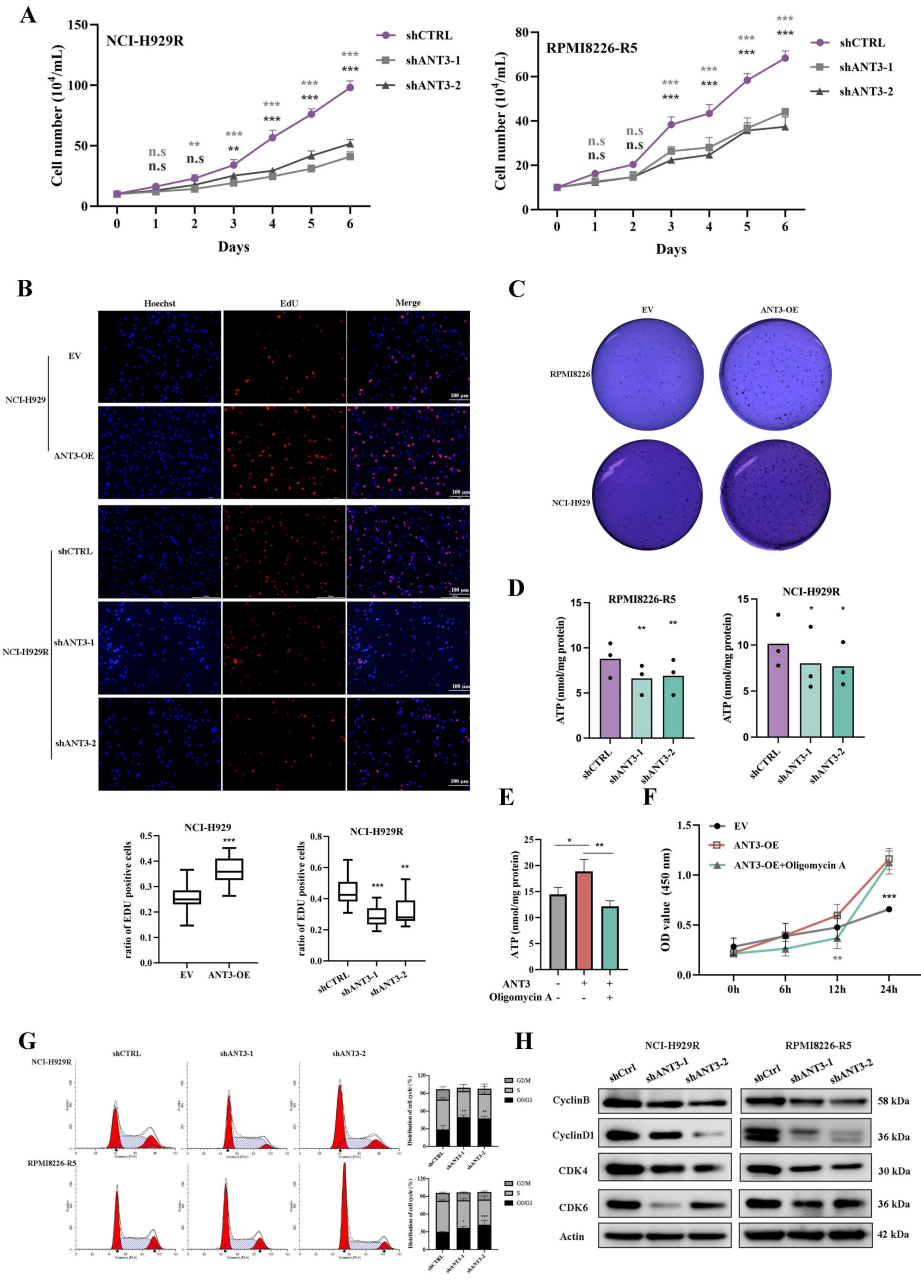
** ANT3 promotes myeloma cell proliferation via mediating intracellular ATP level.** (**A**) The cell numbers of MM stable strain (NCI-H929R, RPMI8226-R5) with ANT3 knockdown or negative control by short hairpin RNA (shANT3/shCTRL), were recorded after staining with trypan blue for 6 consecutive days (n = 3, ** *P* < 0.01, *** *P* < 0.001). (**B**) EdU assay detection of cell proliferation of NCI-H929 and NCI-H929R stably transfected with ANT3-OE/EV or shANT3/shCTRL, respectively. Scale bars =100 μM, original magnification: ×400. The bar graph showed the proportion of Edu positive cells (** *P* < 0.01, *** *P* < 0.001). (**C**) Soft agar colony formation assay of ANT3-OE/EV MM cells (NCI-H929 and RPMI8226). (**D**) Intracellular ATP of shANT3/shCTRL MM cells (NCI-H929R, RPMI8226-R5) were quantified by luminometer (n = 3, * *P* < 0.05, ** *P* < 0.01). (**E-F**) Intracellular ATP and growth curve of OE/EV MM cells treated with oligomycin A (5 μM) were detected (n = 3, ** *P* < 0.05, ** *P* < 0.01, *** *P* < 0.001). (**G**)Flow cytometry detection of cell cycle of NCI-H929R, RPMI8226-R5 stably transfected with shANT3/shCTRL. The right bar graph showed the percentage of cells in each stage (* *P* < 0.05, ** *P* < 0.01). (**H**) Western blot analysis of the expressions of G0/G1-phase-related proteins (Cyclin D1, CDK4, CDK6) and Cyclin B in MM cells (NCI-H929R, RPMI8226-R5) stably transfected with shANT3 /shCTRL. Actin served as a loading control.

**Figure 3:**
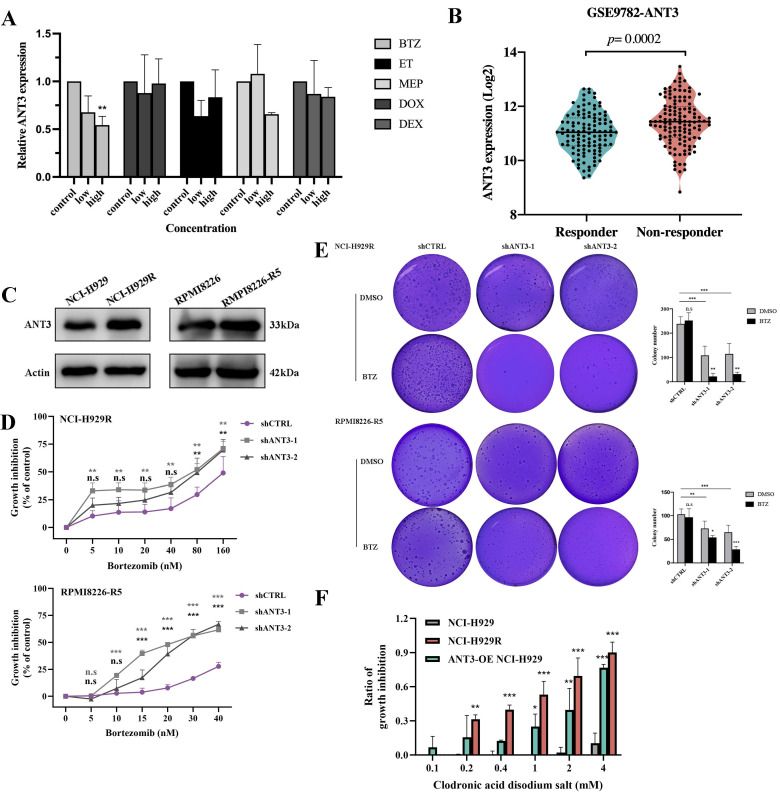
** ANT3 is an important factor in BTZ resistance of myeloma.** (A) qRT-PCR was used to detected the level of ANT3 in MM cells treated with gradient chemotherapy drugs including bortezomib (BTZ), Etoposide (ET), Melphalan (MEP), doxorubicin (DOX), and dexamethasone (DEX), concentration based on their IC_50_ (n = 3, ** *P* < 0.05)_._ (**B**) Analysis of ANT3 expressions in bortezomib responders and non-responders using GEO database (GSE9782). (**C**) Western Blot compared the expression levels of ANT3 between sensitive and corresponding BTZ-resistant MM strains (NCI-H929/NCI-H929R, RPMI8226/RPMI8226-R5). (**D**) Growth inhibition was detected by CCK-8 assay after treating shANT3/shCTRL MM resistant cells (NCI-H929R, RPMI8226-R5) with Bortezomib (n = 3, ** *P* < 0.01, *** *P* < 0.001), and (**E**) demonstrated the soft agar colony formation ability. The right bar graph showed the counting of colony numbers (** *P*<0.01, *** *P*<0.001). (**F**) MM cells were treated with gradient concentrations of clodronic acid disodium salt (0, 0.1, 0.2, 0.4, 1, 2, 4 mM) for 72 h and cell viability was detected by CCK-8 (n = 3, *p < 0.05, **p < 0.01, ***p < 0.001).

To explore the specific role of ANT3 in MM cells, we constructed stably transfected cell lines in which ANT3 was either overexpressed or knocked down. We observed significant changes in the growth rate of MM cells after differential expression of ANT3. By recording the growth curves of ANT3-OE/EV and shANT3/shCTRL MM cells and performing EdU staining, we found that ANT3 was associated with the proliferation of MM cells (**Figure 2A-B; Figure S2A-B**). The soft agar colony formation assay was also performed, and the results showed that the number of cell colonies decreased after ANT3 was knocked down, while ANT3 overexpression showed the opposite trend, further confirming the biological function of ANT3 in cell proliferation (**Figure 2C; Figure 3E**).

Considering that ANT3 mediates ADP/ATP exchange in cells, we initially speculated that ANT3 expression levels alter the intracellular ATP supply, resulting in changes in the cell growth rate. Therefore, intracellular ATP levels were detected and found to decrease in shANT3 MM cells, which preliminarily confirmed our hypothesis (**Figure 2D**). Subsequently, we applied oligomycin A, an ATP synthase inhibitor, to ANT3-OE MM cells and detected intracellular ATP levels and growth rates (**Figure 2E-F**). The results showed that oligomycin A reversed the trend caused by ANT3 overexpression by reducing intracellular ATP levels and restricting the proliferation rate to some extent. These results showed that the effect of ANT3 on proliferation was related to its effect on cellular ATP levels.

The lower levels of intracellular ATP affected the cell cycle process (**Figure S2C**). Furthermore, shANT3 MM cells tended to accumulate in the G0/G1 phase, as shown in Figure 2G. The decrease in the levels of G0/G1 phase-related proteins, such as CDK4, CDK6, and Cyclin D, supported the flow cytometry results (**Figure 2H**). The cell cycle arrest in the G0/G1 phase explains why the knockdown of ANT3 slowed proliferation.

### ANT3 affects the resistance of MM cells to BTZ

**Figure 4:**
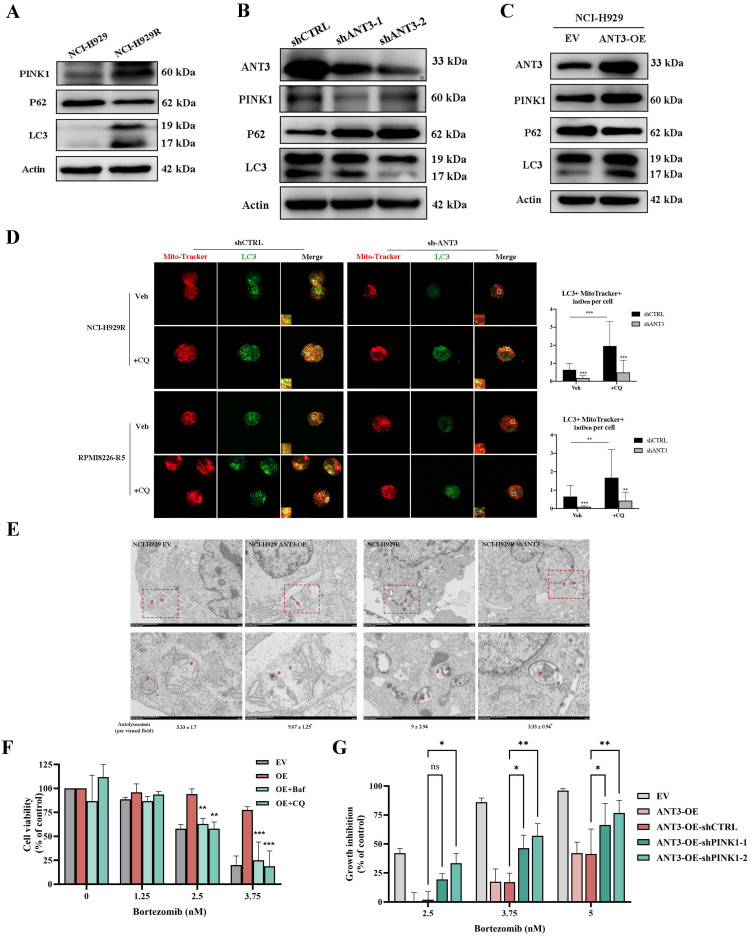
** ANT3 positively regulates mitophagy leading to bortezomib resistance in myeloma cells.** (**A-C**) The markers of mitophagy (PINK1, P62 and LC3) were detected by western blot in three groups of MM cells, including sensitive/resistant cells (NCI-H929/NCI-H929R), shANT3/shCTRL cells (NCI-H929R) and OE/EV MM cells (NCI-H929). (**D**) NCI-H929R and RPMI8226-R5 stably transfected with shANT3/shCTRL were treated with CQ (20 μM) for 2 h in advance and then stained by Mito-tracker (200 nM) for 20 min. The expression and co-localization of LC3 puncta (green) and mitochondria (red) were recorded using laser confocal microscopy. The right bar graph showed the fluorescence intensity of the overlapping part of LC3 puncta and Mito-Tracker per cell (n = 20, ** *P* < 0.01, *** *P* < 0.001). (**E**) Representative transmission electron microscopy images of mitochondria network in OE/EV MM cells (NCI-H929) and shANT3/shCTRL MM resistant cells (NCI-H929R). (**F**) OE/EV MM cells (NCI-H929) were pre-treated with chloroquine diphosphate (CQ, 10 μM) or bafilomycinA1 (Baf, 2 nM) 4 h in advance, and then treated with bortezomib for 48 h. CCK-8 assay was used to evaluate the cell viability. (**G**) ANT3-overexpressed MM cells were treated with bortezomib for 48 h after knocking down PINK1. CCK-8 assay was used to evaluate the cell viability.

Previous microarray results showing altered expression levels of ANT3 in several tumors after chemotherapy have suggested that ANT3 might be related to drug resistance. We then administered common chemotherapeutic drugs for myeloma to MM cells and detected ANT3 expression. ANT3 expression was downregulated in a concentration-dependent manner after BTZ treatment (**Figure 3A**). We then analyzed the differences in ANT3 expression between BTZ responders and non-responders according to the GSE9782 dataset from the GEO database. We compared the differences between BTZ-resistant MM cells and their corresponding sensitive cells (**Figure 3B-C**). The results showed higher ANT3 levels in the non-respondent patients and BTZ-resistant cells, suggesting a potential correlation with drug resistance. We then overexpressed ANT3 in sensitive cells and knocked down ANT3 in BTZ-resistant cells, which were treated with BTZ. As expected, the expression level of ANT3 affected the sensitivity of MM cells to BTZ based on the results of the CCK-8 cell viability assay (**Figure 3D; Figure 4F**). After BTZ treatment, the growth inhibition rate of MM-resistant cells with ANT3 knockdown was significantly higher than that in the control group. Additionally, fewer cell colonies were formed in the shANT3 group after BTZ treatment, which confirmed the relationship between ANT3 expression and BTZ resistance (**Figure 3E**). Most notably, the re-sensitization of BTZ-resistant cells after the knockdown of ANT3 suggests that targeted intervention in ANT3 might provide a new treatment option for clinical BTZ-resistant patients. Therefore, we also treated MM cell lines with the current ANT inhibitor, named clodronic acid disodium salt. The result showed that the toxic effect on MM cells was concentration-dependent (**Figure 3F**). Moreover, compared to the wild-sensitive strain, the resistant strain was more sensitive to clodronate. ANT3-overexpressed cell strain also tended to be more sensitive to clodronate. These results preliminarily confirmed the clinical application value of ANT3 inhibition in BTZ-resistant patients.

### ANT3-PINK1 mediated mitophagy affects BTZ resistance in MM cells

The anti-MM efficacy of many chemotherapeutic drugs is attributed to the induction of oxidative stress and mitochondrial dysfunction-induced cell apoptosis. In contrast, mitophagy maintains homeostasis by rapidly removing damaged mitochondria. Here, we inferred that ANT3 participates in mitophagy activation since ANT3 is located in the inner mitochondrial membrane and plays a role in mitochondrial stability. By measuring several mitophagy activation markers, including LC3B-I/II, P62, and PINK1, via western blot, we detected higher levels of mitophagy in BTZ-resistant and ANT3-OE cells, than in their respective controls (**Figure 4A, C; Figure S3A-B**). However, in shANT3 MM cells, mitophagy levels declined significantly, indicating that ANT3 positively regulates mitophagy in MM cells (**Figure 4B; Figure S3C**). Using chloroquine diphosphate (CQ) to block lysosomal digestion and induce LC3 accumulation, we observed that LC3 puncta accumulation was more evident in NCI-H929R and RPMI8226-R5 cells, and the LC3^+^MitoTracker^+^ fluorescence intensity per cell was significantly stronger than that in shANT3 cells (**Figure 4D**). We also visualized the mitophagy process by taking the fusion of mitochondria with autophagosomes via TEM and quantified autolysosomes per visual field (16 μm^2^). The results further verified that the mitophagy level of the resistant strain was higher than that of the sensitive strain, and that the expression level of ANT3 positively regulated mitophagy in MM (**Figure 4E**).

Subsequently, follow-up experiments were conducted to explore the association between mitophagy and BTZ resistance in MM cells. We treated MM cells (NCI-H929) with BTZ, and detected the mitophagy marker proteins. The results indicated that BTZ effectively reduced mitophagy in MM cells in a concentration-dependent manner (**Figure S4A**). Moreover, MM cells were pretreated with the agonist rapamycin for 4 hours, and then BTZ was added. The results of the CCK-8 assay showed that sensitive MM cells could tolerate higher concentrations of BTZ after rapamycin treatment (**Figure S3D**). In contrast, pretreatment of MM cell-resistant cells with bafilomycinA1 (Baf), a mitophagy inhibitor, re-sensitized NCI-H929R, and RPMI8266-R5 cells to BTZ (**Figure S3E**). These results illustrate that mitophagy affects the response of MM cells to BTZ. To test whether ANT3 affects BTZ resistance in MM cells through mitophagy, we treated MM cells with mitophagy inhibitors (Baf and CQ) and subsequently added gradient BTZ. As shown in Figure 4F, inhibition of mitophagy effectively reversed the BTZ resistance caused by ANT3 overexpression. In addition, PINK1 knockdown partially restored the BTZ response in MM cells affected by ANT3 overexpression (**Figure 4G**). We thus hypothesized that ANT3-PINK1 mediated mitophagy and then affected the response of MM cells to BTZ.

### ANT3-mediated mitophagy is dependent on regulating PINK1 transport and stabilization

**Figure 5:**
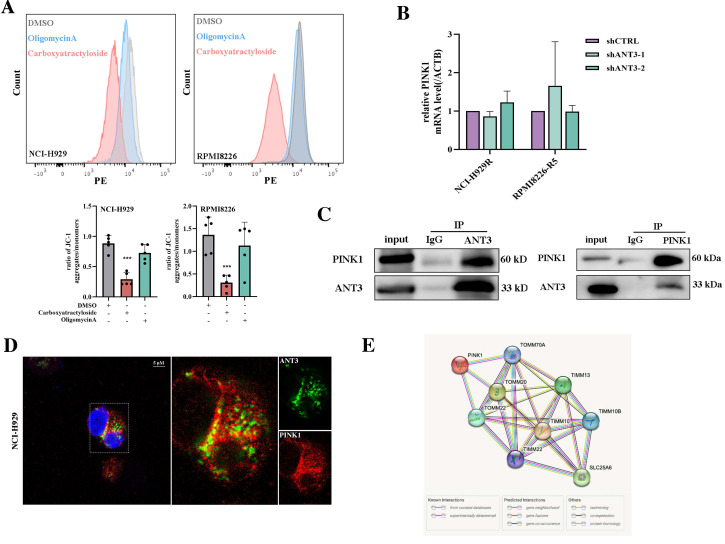
** ANT3 regulates PINK1-mediated mitophagy at the protein level.** (**A**) MM cells (NCI-H929, RPMI8226) were treated with oligomycin A (5 μM) or CTRA (20 μM), and then mitochondrial membrane potential detection reagent (JC-1) was used to detect the level of mitochondrial membrane potential (MMP) by flow cytometry. The bar chart below compared the percentage of PE/FITC fluorescence intensity, representing aggregates and monomers respectively (n = 5, *** *P* < 0.001). (**B**) The relative expression of PINK1 at the transcriptional level was measured by qPCR. (**C**) Endogenous co-immunoprecipitation was conducted in NCI-H929 using anti-ANT3 or anti-PINK1, followed by immunoblotting using anti-ANT3 and anti-PINK1. Anti-IgG was used as a non-specific control. (**D**) MM cell (NCI-H929) was photographed by confocal microscope to detect the colocalization of ANT3 and PINK1. (**E**) Genetic interaction network associated with ANT3 and PINK1 analyzed in STRING. Circle represents node (proteins), while line represents edge (connections).

In the following sections, we describe how ANT3 regulates mitophagy. We first considered the biological function of ANT3 as an ADP/ATP transferase that affects the functions of mitochondria including mitochondrial superoxide level and calcium level, leading to cellular stress and ultimately mitophagy (**Figure S5A-B**). Thus, we applied carboxyatractyloside or oligomycin A to simulate the inhibition of ANT3's function and then detected MMP levels using JC-1 staining. The result showed a decrease or no significant change in MMP, indicating membrane depolarization and representing the early stage of mitophagy (**Figure 5A**). This result contradicted our previous conclusion that ANT3 positively regulates mitophagy, suggesting that ANT3 mediates mitophagy via an independent and unknown pathway.

To further explore the regulatory mechanism of ANT3 in mitophagy, we first examined the effects of ANT3 on PINK1, which is the key protein involved in the occurrence of mitophagy. We considered that the regulation of PINK1 by ANT3 occurred at the protein level because the qPCR results did not show a statistical difference in PINK1 mRNA levels between the shCTRL and shANT3 groups (**Figure 5B**). The interaction between ANT3 and PINK1 at the protein level was also examined in MM cells by co-immunoprecipitation (**Figure 5C-D**). We then set ANT3 and PINK1 as nodes, and established an interaction network using the STRING database (https://string-db.org/). According to this network analysis, almost all proteins connecting ANT3 and PINK1, including Tomm20, Timm10, and Timm22, were mitochondrial inner and outer membrane transporters, suggesting that ANT3 participate in the transportation process of PINK1 (**Figure 5E**).

**Figure 6:**
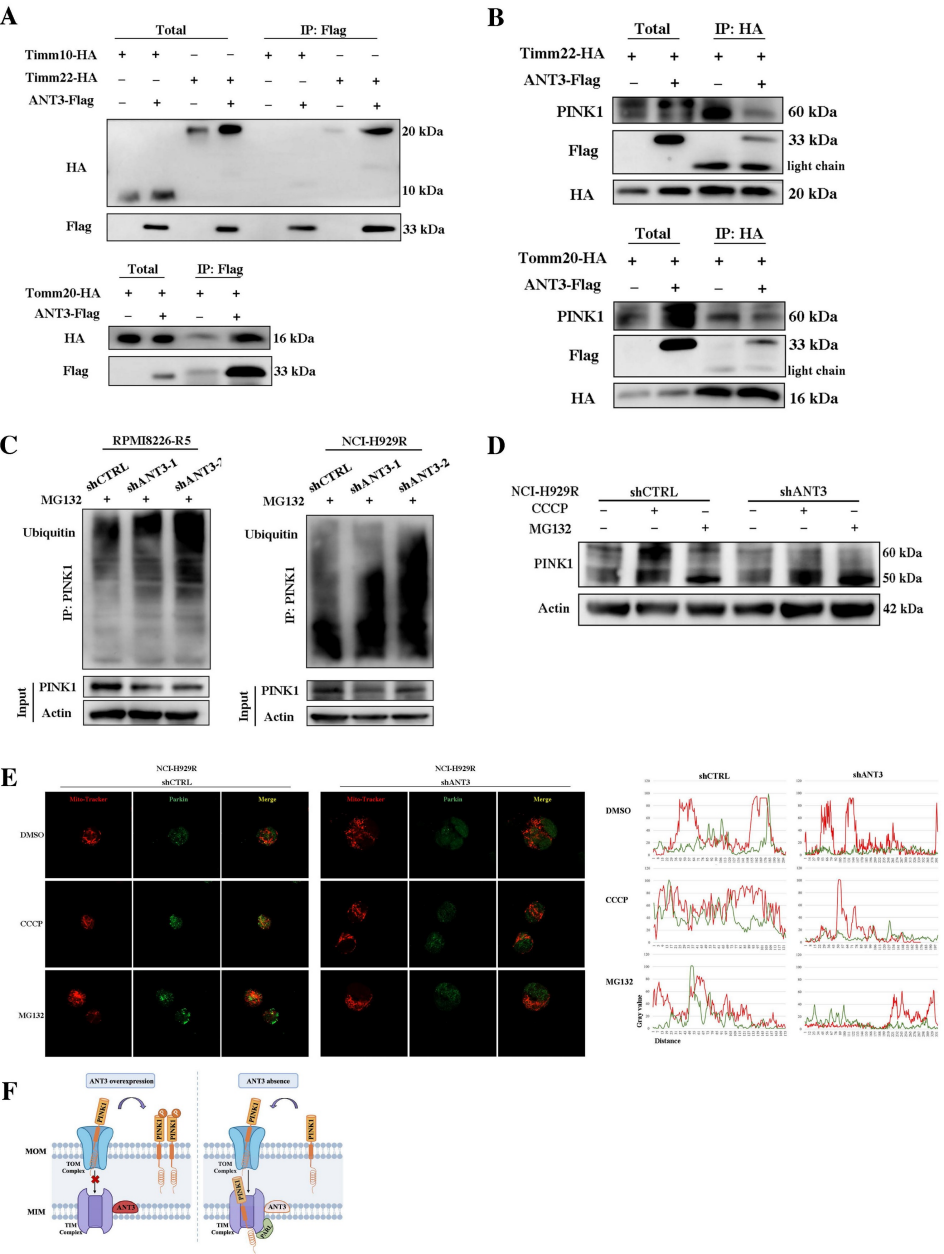
** ANT3 interacts with Timm22 to mediate PINK1 transport and stabilization.** (**A**) HEK293T cells were transfected with ANT3-Flag, Timm10-HA /Timm22-HA /Tomm20-HA, or both plasmids. Cell lysates were immunoprecipitated using anti-Flag and the immunoprecipitants or input were analyzed by immunoblotting with anti-HA and anti-Flag. (**B**) MM cells (ARP-1) were transfected with Timm22-HA /Tomm20-HA alone or in combination with ANT3-Flag. Cell lysates were immunoprecipitated using anti-HA and the immunoprecipitants or input were analyzed by immunoblotting with anti-HA, anti-Flag and anti-PINK1. (**C**) Knockdown of ANT3 in MM cells increased accumulation of polyubiquitinated PINK1 when treated with MG132. PINK1 was pulled down and anti-ubiquitin antibody was used to detect polyubiquitinated PINK1. (**D**) MM resistant cells stably transfected with shANT3/shCTRL (NCI-H929R) were treated with DMSO, CCCP (10 μM) or MG132 (10 μM) for 4 h and the expression of PINK1 was detected by Western blot. (**E**) After the pre-treatment of CCCP or MG132, shANT3/shCTRL MM cells were stained by Mito-Tracker (red) and anti-Parkin (green). The level of recruitment of Parkin to mitochondria were recorded using laser confocal microscopy. (**F**) Model of ANT3-mediated PINK1 transport via interaction with Timm22 and Tomm20.

Then we labeled these transporters with the HA tag, detected the interaction between ANT3 and TOM/TIM proteins in HEK293T cells, and selected Tomm20 and Timm22 for subsequent examination according to the positive Co-IP results (**Figure 6A**). Since the transport of PINK1 on the mitochondrial membrane requires the involvement of transporter proteins under physiological conditions, we next detected the changes in the interaction level between PINK1 and Tomm20 or Timm22 after ANT3 overexpressing. Although the level of PINK1 in MM cells increased after ANT3 overexpression, the interaction between PINK1 and Timm22 was significantly weakened, whereas that interaction between PINK1 and Tomm20 did not show any obvious difference (**Figure 6B**).

This result indicated that the expression level of ANT3 mainly affected the transfer and stability of PINK1 to the inner membrane, in which case PINK1 could not be cleaved into 50 kDa fragments or degraded. To verify this result, we applied CCCP and MG132 to stabilize the 60-kDa full-length form and the 50-kDa fragment of PINK1, respectively, and then detected PINK1 expression in shANT3 and shCTRL MM cells. We used an 8% gel to separate the two strips and observed that more 50-kDa fragments were formed in shANT3 cells, and more 60-kDa full-length PINK1 was formed in the control group (**Figure 6D**). Due to the fact that PINK1 is cleaved after being transferred to the inner membrane and then released into the cytoplasm for hydrolysis through the ubiquitin-proteasome pathway, we also detected changes in the ubiquitination level of PINK1 after changes in ANT3 expression levels. The results showed a significant increase in the level of PINK1 ubiquitination modification in shANT3 MM cells, suggesting that PINK1 underwent increased ubiquitin-proteasome hydrolysis (**Figure 6C**).

Furthermore, to confirm whether only full-length PINK1 stabilized in the outer membrane could exert the function of recruiting Parkin, we also determined the recruitment level of Parkin after treatment with CCCP and MG132 (**Figure 6E**). The images obtained using laser scanning confocal microscopy showed that only full-length PINK1 could effectively recruit Parkin to the mitochondria, whereas the accumulation of 50-kDa PINK1 by MG132 could not effectively activate mitophagy. Notably, there was no obvious Parkin accumulation or recruitment in shANT3 cells, even in the presence of CCCP. The complete process of ANT3 mediating PINK1 transport process is shown in the model diagram (**Figure 6F**). Under pathological conditions such as drug resistance, the aberrant high expression of ANT3 interferes with the binding of PINK1 to Timm22, thereby promoting the stabilization of PINK1 in the outer membrane and leading to mitophagy. After the intervention, ANT3 was absent, and PINK1 would be transported to the inner mitochondrial membrane and cleaved, limiting PINK1-mediated mitophagy.

### Knocking down ANT3 enhances BTZ sensitivity and inhibits tumor growth *in vivo*

**Figure 7:**
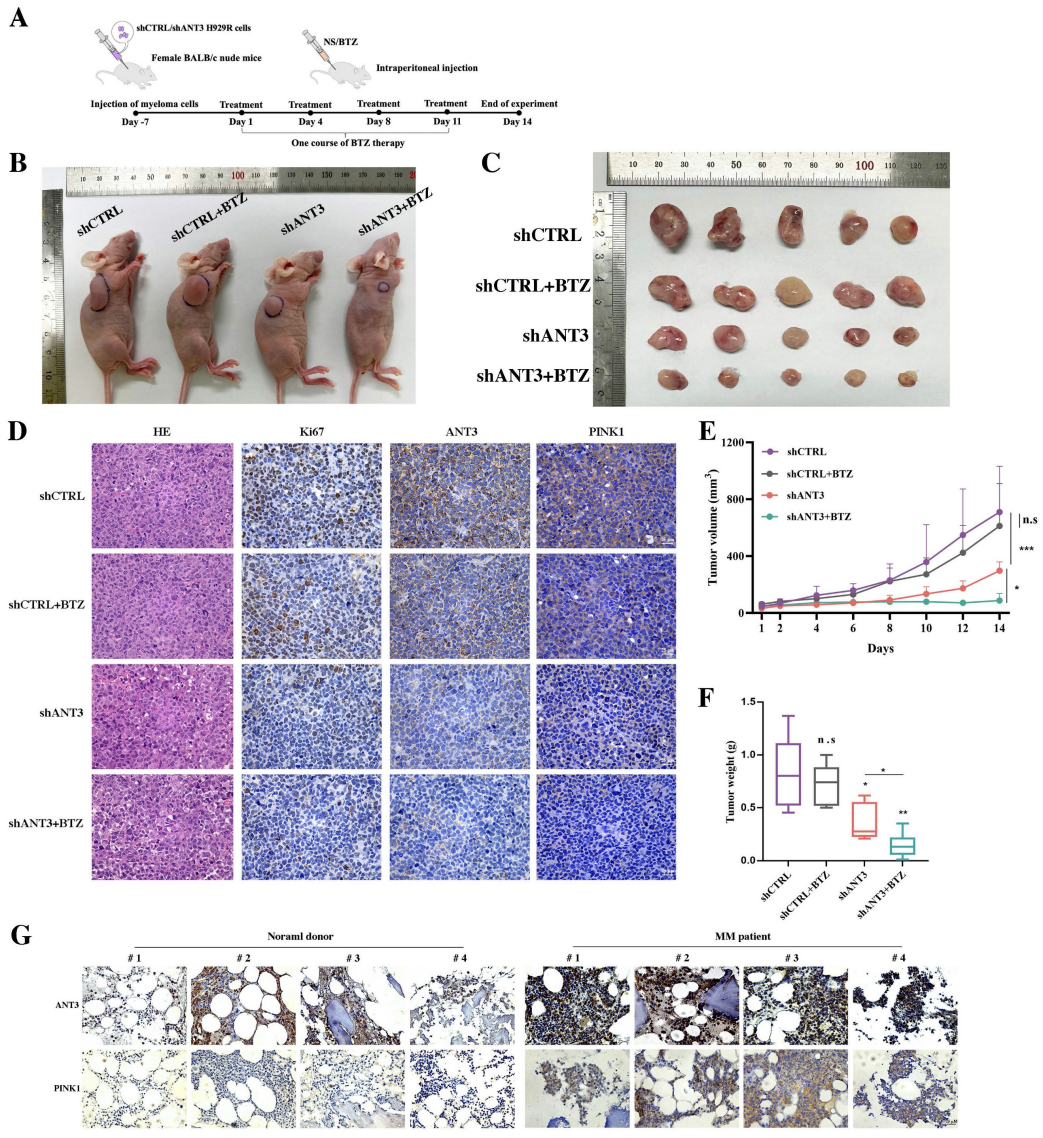
** Knocking down ANT3 inhibits the growth of MM transplanted tumors and promotes sensitivity to BTZ *in vivo*.** (**A**) Diagram of the establishment of subcutaneous xenograft tumors and BTZ treatment process. (**B-C**) Nude mice bearing MM (shANT3/shCTRL NCI-H929R) were treated with normal saline or bortezomib. Gross appearance of tumor samples in each group (n=5 mice/group). (**D**) Tumor tissues of each group were stained with HE and IHC analysis including Ki67, ANT3 and PINK1. Scale bars = 25 μM, original magnification: ×400. (**E-F**) Tumor volumes during the experiment were recorded and so did the weight of tumors at the end of the experiment (n=5, * *P* < 0.05, ** *P* < 0.01, *** *P* < 0.001). (**G**) Bone marrow slice specimens from four normal donors and four myeloma patients were detected indicators including ANT3 and PINK1 by IHC.

To further investigate the effect on the response to BTZ after knocking down ANT3 in MM, we established a mouse MM xenograft model via the subcutaneous injection of NCI-H929R cells. The tumor volume and weight differences showed that the shANT3 group exhibited a decreased proliferation rate (**Figure 7B-C; E-F**). Moreover, we intraperitoneally injected BTZ (1 mg/kg) into the treatment group on the 1st, 4th, 8th, and 11th days of the experiment, according to the clinical course of BTZ (**Figure 7A**). Consistent with the *in vitro* findings*,* the shANT3 group showed greater sensitivity to BTZ than the control group, regardless of the final tumor volume or weight. Moreover, immunohistochemistry (IHC) revealed that the shANT3 group had lower levels of the proliferation marker Ki-67 and mitophagy markers in the tumor tissues (**Figure 7D**). To explore the clinical significance of ANT3 and mitophagy markers in MM, we collected eight bone marrow biopsy specimens from patients with relapsed and drug-resistant MM, or people with non-malignancy. The results showed that the expression of ANT3 and PINK1 in the bone marrow biopsy specimens of MM patients was significantly higher than that in the control specimens (**Figure 7G**), indicating that detecting the levels of ANT3 and mitophagy is of great significance for guiding MM clinical treatment strategy, especially in relapsed and drug-resistant MM patients. These results confirmed that shANT3 improved the response to BTZ in MM *in vivo*.

## Discussion

Bortezomib is the first and still the most widely used proteasome inhibitor in myeloma treatment, and it has dramatically improved the prognosis of patients with MM. However, MM remains incurable mainly because of inevitable clinical resistance. It is critical to elucidate the underlying mechanisms of primary or acquired resistance and to identify key molecular targets, based on which targeted drugs alone or combined with first-line chemotherapy drugs may have better therapeutic effects.

Here, we identified one protein, ANT3, in TRIP13-interacting proteins during previous study, which is one of the chromosomal instability genes, using liquid chromatography-tandem mass spectrometry (LC-MS/MS)^22^. The biological function of the ANT family is related to intracellular energy metabolism and apoptosis, and aberrant expression of ANT could affect the normal growth of cells^10^, ^23^.

Interestingly, ANT3 overexpression in HeLa cells (human cervical cancer cells) was found to promote apoptosis, whereas in Jurkat cells (acute T-cell leukemia cells), ANT3 showed anti-apoptotic effects. The effects of ANT3 on apoptosis in different cell lines suggest that the function of ANT3 may be highly cell-type-dependent^13^, ^14^. Analysis of ANT3 expression levels in 33 common human cancers also showed that ANT3 is highly expressed in only a few cancer types, including hematological malignancies, compared to normal tissues. Additionally, there is little evidence demonstrating its relationship with cancer development or drug resistance, except for its potential as a diagnosis biomarker^12^. Here, we found for the first time that ANT3 was aberrantly highly expressed in MM and correlated with rapid progression and poor prognosis in the orthotopic MM model. The expression level of ANT3 was related to higher risks, including β2M, the presence of cytogenetic abnormalities, and the late period of the ISS system, showing the value of ANT3 in judging prognosis for patients with myeloma. In this study, we confirmed the growth-promoting effect of ANT3 on MM and showed that ANT3's role in ADP/ATP translocation could help explain this phenotype. In cancer cells, cell cycle transitions are sensitive to the amount of available ATP^24^, ^25^. After ANT3 was knocked down, the intracellular ATP level decreased, which in turn affected the expression level of cell cycle checkpoint proteins that regulate the transition from the G0 to the G1 phase, thereby interfering with cell proliferation.

The proliferative phenotype can be explained by the biological function of ANT3, while it can hardly explain the mitophagy phenotype we discovered. By detecting the expression levels of mitophagy marker proteins using western blotting, immunofluorescence to detect the co-localization of mitochondria and LC3, and TEM to detect the occurrence of mitophagy visually after the overexpression or knockdown of ANT3, we preliminarily confirmed that ANT3 positively regulated mitophagy levels. However, the inhibition of ANT function as an ADP/ATP translocase accelerated mitophagy, indicating that ANT3 promoted PINK1-mediated mitophagy independently. Increasing evidence has shown the crucial role of mitophagy in cancer progression and drug resistance^26^, ^27^. PINK1, a serine/threonine kinase, is ordinarily transferred to the inner membrane of mitochondria through the translocase of the outer/inner mitochondrial membrane under physiological conditions and is subsequently degraded by presenilin-associated rhomboid-like protein (PARL)^28^, ^29^. When the MMP is damaged, PINK1 stabilizes in the outer mitochondrial membrane, where it recruits Parkin, Ub, and P62, resulting in mitophagy^30^, ^31^. Here, we identified proteins that may interact with ANT3 and PINK1 and validated them using Co-IP. We found that high ANT3 expression hindered Timm22 from binding and stabilizing PINK1 in the inner membrane, in which case PINK1 could not be cleaved or degraded under the usual procedure. A previous study showed that members of the ANT family interact with Timm23 and mediate PINK translocation^7^. Taken together, our findings regarding the mechanism of ANT3 mediating mitophagy differed from those of the aforementioned studies. Using CCCP to reduce MMP or MG132 inhibiting proteasome, laser scanning confocal microscopy showed that CCCP enhanced the recruitment of Parkin to the mitochondria, and this trend was more pronounced in the shCTRL group than in the shANT3 group. Moreover, the accumulation of the 50-kDa fragment of PINK1 by MG132 did not contribute to the recruitment of Parkin. It is valid to consider that the overexpression of ANT3 led to PINK1 being in the outer membrane in its full-length form, thereby promoting mitophagy.

Interestingly, the restriction of PINK1-Parkin-mediated mitophagy by ANT3 knockdown increased the sensitivity of MM cells to BTZ, as controversy still exists over whether mitophagy benefits cancer development. Currently, mitophagy is believed to help tumor cells adapt to the pressure of chemotherapy^32^. Our results showed increased mitophagy in the BTZ-resistant strains (NCI-H929R and RPMI8226-R5) and ANT3-OE MM cells. After ANT3 was knocked down, the level of mitophagy decreased, and sensitivity to BTZ in resistant strains was restored. This trend was further verified in a mouse model. Records of tumor volume and Ki-67 expression showed lower levels of tumor growth in the shANT3 group than in the control group, and BTZ treatment nearly halted shANT3 MM growth. Most drugs used in MM induce cell apoptosis by causing mitochondrial dysfunction. In contrast, mitophagy rapidly removes damaged mitochondria to maintain cellular homeostasis, which may explain why increased mitophagy enhances the effects of BTZ. We also applied mitophagy inducers and inhibitors to treat BTZ-sensitive and BTZ-resistant myeloma strains respectively. We found that activating mitophagy could render sensitive strains resistant, whereas inhibiting mitophagy could restore sensitivity of the resistant strains. This conclusion is in line with the aforementioned speculation. We also measured the mitophagy levels in tumor tissues using IHC. ANT3 knockdown restricted mitophagy *in vitro*, and BTZ had similar effects, further verified by detecting mitophagy levels after gradient BTZ treatment of MM cells using western blotting. Moreover, we collected and analyzed bone marrow samples from patients with myeloma and control samples from patients with non-hematological malignancies using IHC. Sections from patients with myeloma showed stronger expression of ANT3 and mitophagy levels, consistent with our previous results.

In this study, we investigated the regulatory roles and molecular mechanisms of ANT3 in the occurrence, development, and drug resistance in MM. Initially, public databases, primary patient specimens, and animal models indicated a positive correlation between ANT3, MM progression, and BTZ resistance. We also found that ANT3 promoted the proliferation of MM cells and that this function was associated with the mediation of ATP transport, thereby affecting the cell cycle. In addition, ANT3 promoted BTZ resistance in MM cells, and after ANT3 was knocked down, the resistant cell lines became desensitized, which was related to the induction of mitophagy. Mechanistically, ANT3 interacted with the mitochondrial membrane transporter protein Timm22 to affect PINK1 transport to the inner membrane, stabilizing it in the outer membrane and promoting mitophagy. Our findings indicate that targeting ANT3 is a promising approach to overcome BTZ resistance in patients with myeloma.

## Conclusion

Collectively, our results demonstrate for the first time that ANT3 promotes myeloma progression and BTZ resistance and is associated with poor prognosis. The biological function of ANT3 in ADP/ATP translocation and its novel mechanism of mediating PINK1 stabilization may explain its role in cell proliferation and BTZ resistance, respectively. Our work also provides proof-of-concept in a mouse model that targeted intervention with ANT3 in combination with BTZ may overcome clinical resistance.

## Supplementary Material

Supplementary figures and tables.

## Figures and Tables

**Figure 8:**
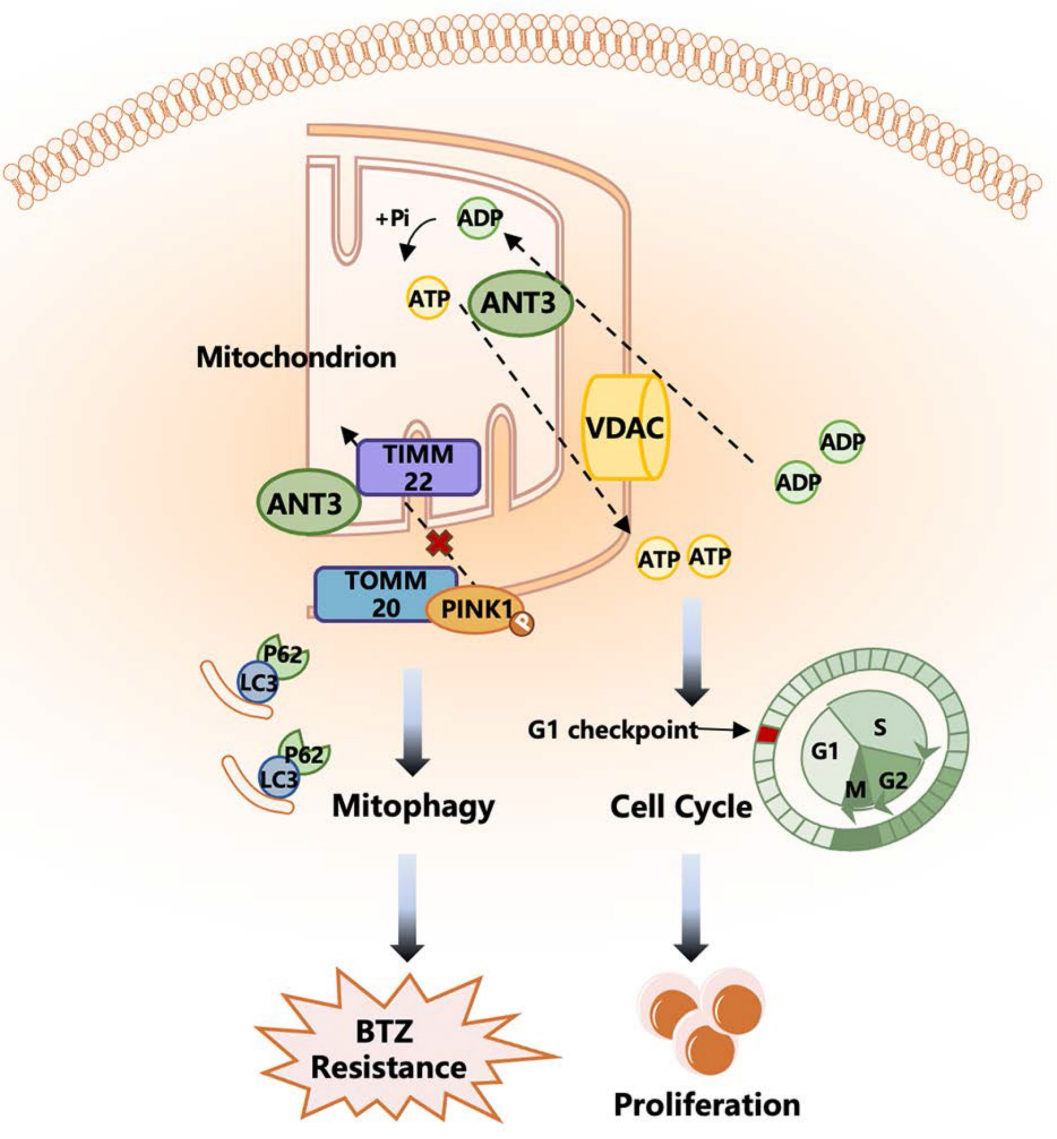
** A working model depicting the major molecular mechanisms of the ANT3 promoting proliferation and BTZ resistance in MM.** ANT3 acts as an important factor in myeloma progression and bortezomib resistance. ANT3 participates in regulating cellular ATP level as ADP/ATP translocase and thus affects the operation of the cell cycle. ANT3 promotes mitophagy independent of its biological function. The interaction with Timm22 blocks the transportation of PINK1 to the inner membrane of mitochondria and stabilizes PINK1 in the outer membrane, which recruits Parkin, LC3, P62 stimulating mitophagy, and finally induces resistance to bortezomib.

**Table 1:** Correlations between clinical biochemical features and ANT3 expression.

Parameters	Group	ANT3 expression			*p*-value
		Cases	Low n (%)	High n (%)	
Gender	Female	57	26 (45.6)	31 (54.4)	0.4166
	Male	113	59 (52.2)	54 (47.8)	
Age	<65 years	128	73 (57.0)	55 (43.0)	0.0014^*^
	≥65 years	42	12 (28.6)	30 (71.4)	
Race	White/Caucasian	155	80 (51.6)	75 (48.4)	0.1764
	Other	15	5 (33.3)	10 (66.7)	
Albumin(g/dL)	<3.5	32	17 (53.1)	15 (46.9)	0.6948
	≥3.5	138	68 (49.3)	70 (50.7)	
β2M (mg/L)	<3.5	64	40 (62.5)	24 (37.5)	0.0393^*^
	≥3.5&<5.5	48	21 (43.8)	27 (56.2)	
	≥5.5	58	24 (42.5)	34 (57.5)	
LDH (U/L)	<190	141	72 (51.1)	69 (48.9)	0.5407
	≥190	29	13 (44.8)	16 (55.2)	
Cytogenetic abnormalities	Yes	38	12 (31.6)	26 (68.4)	0.0100^**^
	No	132	73 (55.3)	59 (44.7)	
ISS	I	55	36 (65.5)	19 (34.5)	0.0199^*^
	II	57	25 (43.9)	32 (56.1)	
	III	58	24 (41.4)	34 (58.6)	

Cytogenetic abnormalities include Del (13), Del (17), t (14; 16), t (14: 20). *p*-values represent a comparison between groups, not against the overall population.

## Abbreviations

MM: Multiple myeloma
BTZ: Bortezomib
ANT3: adenine nucleotide translocase 3
MGUS: monoclonal gammopathy of undermined significance
PINK1: PTEN-induced putative kinase 1
MMP: mitochondrial membrane potential
PBMC: peripheral blood mononuclear cell
EdU: 5-ethynyl-2′-deoxyuridine
TEM: Transmission electron microscopy
LC-MS/MS: liquid chromatography/tandem mass spectrometry
