# Supplementary material for: Aberrant activation of adenine nucleotide translocase 3 promotes progression and chemoresistance in multiple myeloma dependent on PINK1 transport
Source: Int J Biol Sci. 2025 Jan 1;21(1):233–50. doi: 10.7150/ijbs.101850 (PMC11667807; doi:10.7150/ijbs.101850)
Supplement: Supplementary file 1 — Supplementary figures and tables. [file ijbsv21p0233s1.pdf]

Supplementary Tables

Table S1. The sequences of shRNA

Plasmid	Sequence
shANT3-1	CCGGGACCATCAACCTTCGAGAAATCTCGAGATTTCTCGAAGGTTGATGGTCTTTTTG
shANT3-2	CCGGTTCGTGTACCCGCTGGATTTCCTCGAGGAAATCCAGCGGGTACACGAATTTTTG

Table S2. The sequences of primers used in qRT-PCR

Gene	Sequence
PINK1	Forward: 5′-CCCAAGCAACTAGCCCCTC-3′
	Reverse: 5′-GGCAGCACATCAGGGTAGTC-3′
ANT3	Forward: 5′-CAGCGGACGTGGGAAAGTC-3′
	Reverse: 5′-TTGGCCGTATCGTACACGC-3′
GAPDH	Forward: 5′-GGAGCGAGATCCCTCCAAAAT-3′
	Reverse: 5′-GGCTGTTGTCATACTTCTCATGG-3′

Supplementary Figures

Figure S1 The correlation between the expression of ANT3 and tumor. (A) ANT3 expression levels in more than 1000 tumor cell lines from 24 tissues as well as in noncancerous cell line using CCLE database. **(B)** The expression of ANT3 in tumor and normal control using GEPIA2, which contains 33 common human cancer types. The right bar graph showed the optimized comparison in four types of tumors, including diffuse large B cell lymphoma (DLBCL), liver hepatocellular carcinoma (LIHC), testicular germ cell tumor (TGCT), and thymoma (THYM), * $P < 0.05$. **(C)** Serum was extracted from orthotopic mice models, and the concentration of β2-microglobulin (β2-MG), lactic dehydrogenase (LDH), and alkaline phosphatase (ALP) were detected at the end of the experiment, * $P < 0.05$.

Figure S2 The expression level of ANT3 affects the growth rate in myeloma cells. (A) The cell numbers of ANT3 OE/EV MM stable strain (NCI-H929, RPMI8226) were recorded for 6 consecutive days after staining with trypan blue (n=3, ** P<0.01, *** P<0.001). **(B)** EdU assay detection of cell proliferation of RPMI8226 and RPMI8226-R5 stably transfected with ANT3-OE/EV or shANT3/shCTRL, respectively. Scale bars =100 μM, Original magnification: ×400. The bar graph showed the proportion of EdU positive cells (* P < 0.05, ** P<0.01, *** P<0.001). **(C)** MM cells were treated with DMSO and oligomycin A (10 μM), respectively. And then cell cycle was detected by flow cytometry. The right bar graph showed the percentage of cells in each stage (n=3, ** P < 0.01).

Figure S3 The relationship among ANT3 expression, mitophagy and bortezomib resistance in MM. (A-C) The markers of mitophagy (PINK1, P62 and LC3) were detected by western blot in three groups of MM cells, including sensitive/resistant cells (RPMI8226/RPMI8226-R5), shANT3/shCTRL cells (RPMI8226-R5) and OE/EV MM cells (RPMI8226). **(D)** MM sensitive cells (NCI-H929, RPMI8226) were treated with rapamycin

(100 nM) 4 h in advance, and then bortezomib was added in. CCK-8 assay was used to measure cell viability after 48h (n=3, ** $P < 0.01$, *** $P < 0.001$). **(E)** MM resistant cells (NCI-H929R, RPMI8226-R5) were pre-treated with bafilomycin (2 nM) 4 h in advance and bortezomib for 48 h. CCK-8 assay was used to measure cell viability (n=3, ** $P < 0.01$, *** $P < 0.001$).

Figure S4 Mitophagy level after bortezomib treatment. (A) NCI-H929 was treated with bortezomib (0, 2.5, 5 nM) for 48 h, and then mitophagy markers (PINK1, P62, and LC3) were detected by western blot. **(B)** NCI-H929R stably transfected with shANT3 /shCTRL were treated with bortezomib or not, and then the expression of ANT3 and mitophagy level were detected using western blot.

Figure S5. The effect of ANT3 on the mitochondria functions. (A) Knocking down ANT3 triggered mitochondrial Ca2^{+} increase in MM cells (n=3, **$P < 0.01$, *** $P < 0.001$). **(B)** Flow cytometry was used to determine induction of mitochondrial superoxide in shANT3/shCTRL MM cells. The right bar graph showed the percentage of the cells of increased superoxide in each group (n=3, *** $P < 0.001$, **** $P < 0.0001$).

Figure S1

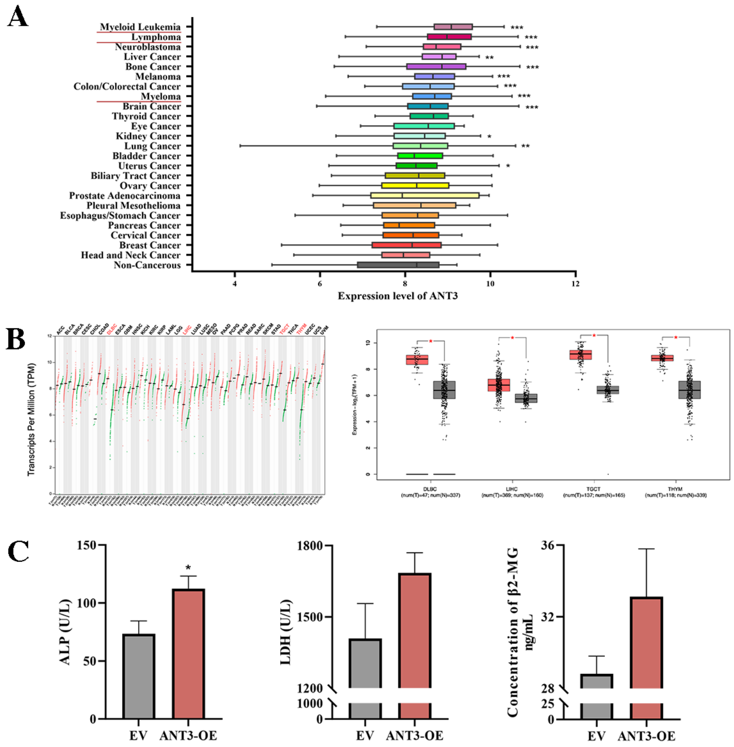

Figure S2

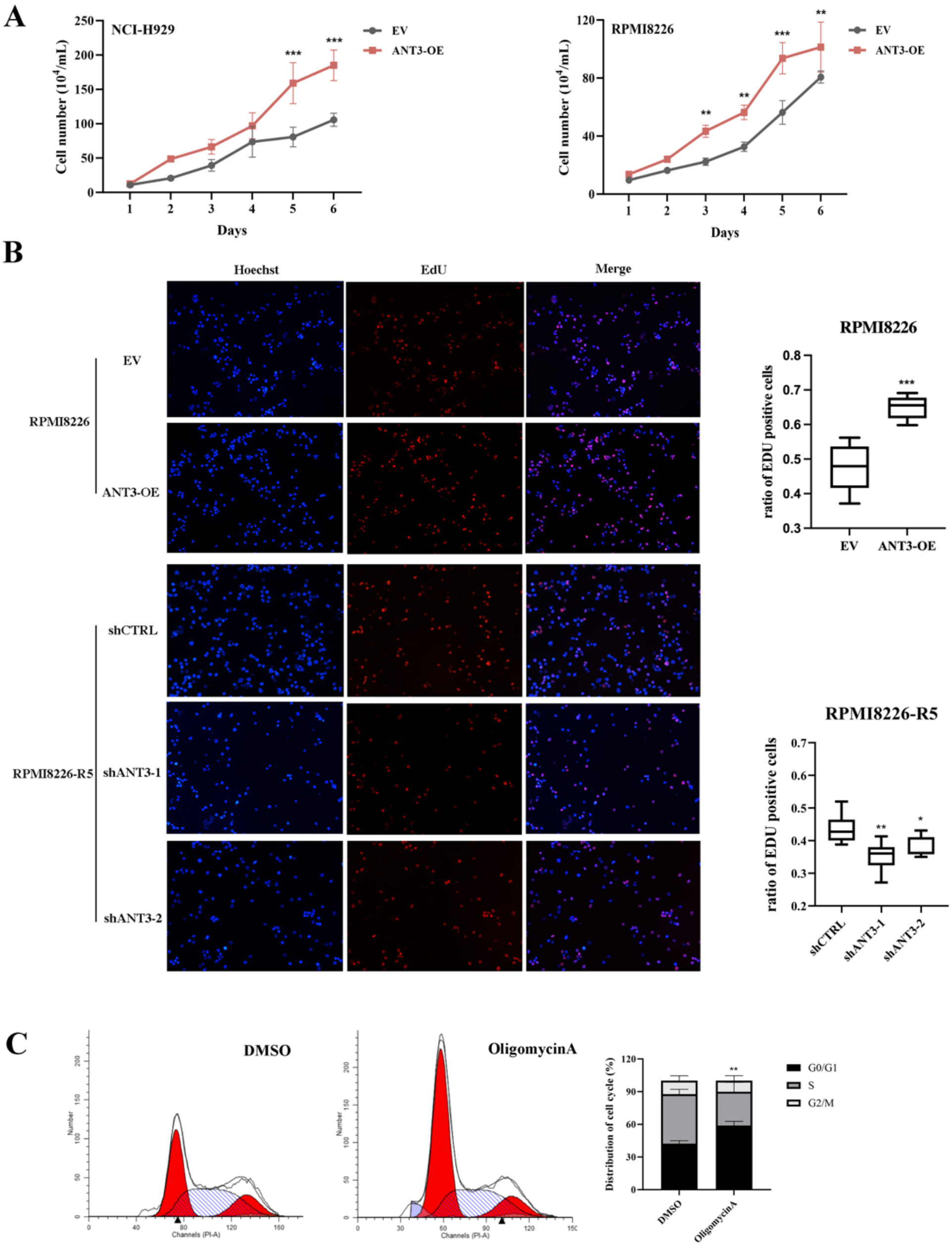
A
NCI-H929
EV
ANT3-OE
Cell number (10⁴/mL)
Days
RPMI8226
B
Hoechst
EdU
Merge
RPMI8226
EV
ANT3-OE
RPMI8226-R5
shCTRL
shANT3-1
shANT3-2
ratio of EDU positive cells
C
DMSO
OligomycinA
Distribution of cell cycle (%)
G0/G1
S
G2/M

Figure S3

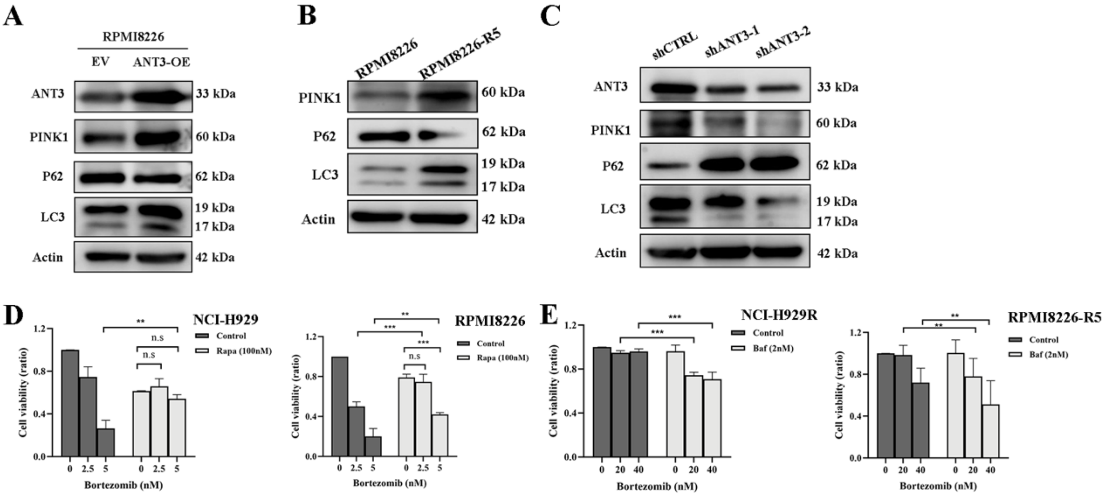

A
RPMI8226
EV
ANT3-OE
ANT3
PINK1
P62
LC3
Actin
33 kDa
60 kDa
62 kDa
19 kDa
17 kDa
42 kDa
B
RPMI8226
RPMI8226-R5
PINK1
P62
LC3
Actin
60 kDa
62 kDa
19 kDa
17 kDa
42 kDa
C
shCTRL
shANT3-1
shANT3-2
ANT3
PINK1
P62
LC3
Actin
33 kDa
60 kDa
62 kDa
19 kDa
17 kDa
42 kDa
D
NCI-H929
Control
Rapa (100nM)
Cell viability (ratio)
Bortezomib (nM)
RPMI8226
Control
Rapa (100nM)
Cell viability (ratio)
Bortezomib (nM)
E
NCI-H929R
Control
Baf (2nM)
Cell viability (ratio)
Bortezomib (nM)
RPMI8226-R5
Control
Baf (2nM)
Cell viability (ratio)
Bortezomib (nM)

Figure S4

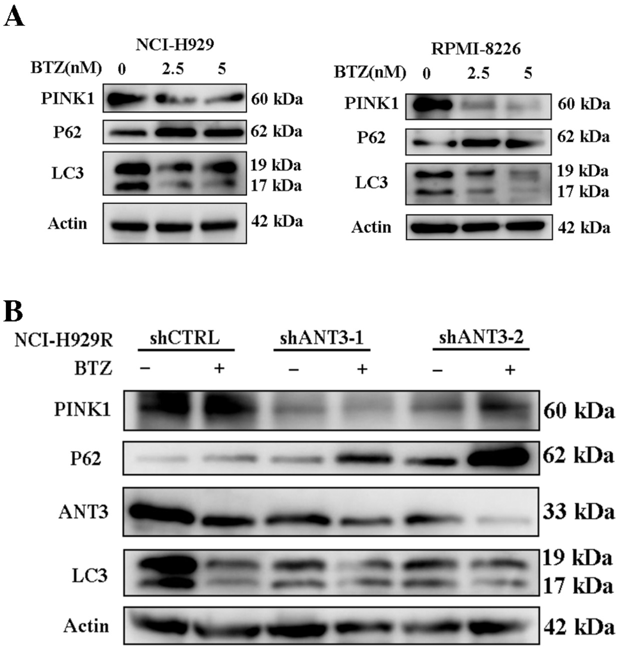
A
NCI-H929
BTZ(nM) 0 2.5 5
PINK1
P62
LC3
Actin
60 kDa
62 kDa
19 kDa
17 kDa
42 kDa
RPMI-8226
BTZ(nM) 0 2.5 5
PINK1
P62
LC3
Actin
60 kDa
62 kDa
19 kDa
17 kDa
42 kDa
B
NCI-H929R
shCTRL
shANT3-1
shANT3-2
BTZ − + − + − +
PINK1
P62
ANT3
LC3
Actin
60 kDa
62 kDa
33 kDa
19 kDa
17 kDa
42 kDa

Figure S5

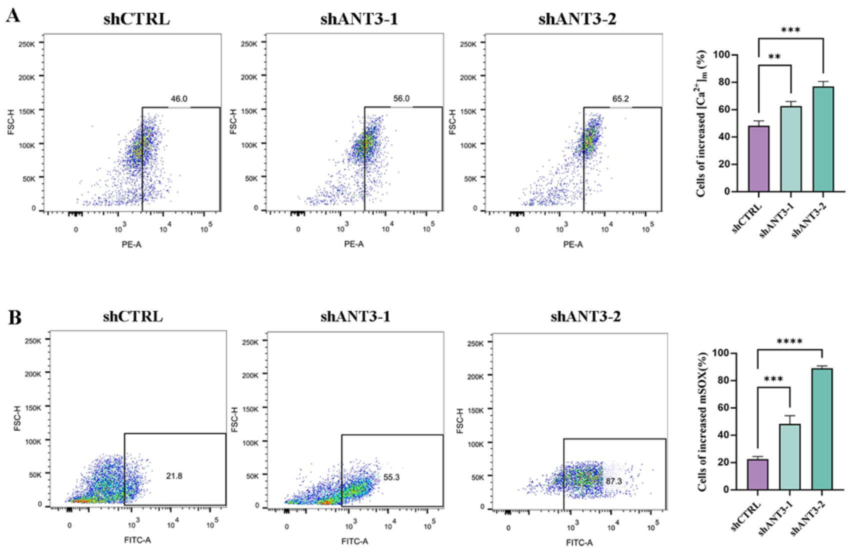
A
shCTRL
shANT3-1
shANT3-2
46.0
56.0
65.2
FSC-H
PE-A
Cells of increased $[Ca^{2+}]_m$ (%)
**
***
B
shCTRL
shANT3-1
shANT3-2
21.8
55.3
87.3
FSC-H
FITC-A
Cells of increased mSOX(%)
***
****
